# A Bayesian Hyperparameter Inference for Radon-Transformed Image Reconstruction

**DOI:** 10.1155/2011/870252

**Published:** 2011-10-30

**Authors:** Hayaru Shouno, Madomi Yamasaki, Masato Okada

**Affiliations:** ^1^Department of Informatics, Graduate School of Informatics and Engineering, University of Electro-Communications, Chofugaoka 1-5-1, Chofu, Tokyo 182-8585, Japan; ^2^Division of Transdisciplinary Science, Graduate School of Frontier Sciences, The University of Tokyo, 5-1-5 Kashiwanoha, Kashiwa, Chiba 277-8561, Japan; ^3^Okanoya Emotional Information Project, RIKEN Brain Science Institute (BSI), 2-1 Hirosawa, Wako, Saitama 351-0198, Japan

## Abstract

We develop a hyperparameter inference method for image reconstruction from Radon transform which often appears in the computed tomography, in the manner of Bayesian inference. Hyperparameters are often introduced in Bayesian inference to control the strength ratio between prior information and the fidelity to the observation. Since the quality of the reconstructed image is controlled by the estimation accuracy of these hyperparameters, we apply Bayesian inference into the filtered back-projection (FBP) reconstruction method with hyperparameters inference and demonstrate that the estimated hyperparameters can adapt to the noise level in the observation automatically. In the computer simulation, at first, we show that our algorithm works well in the model framework environment, that is, observation noise is an additive white Gaussian noise case. Then, we also show that our algorithm works well in the more realistic environment, that is, observation noise is Poissonian noise case. After that, we demonstrate an application for the real chest CT image reconstruction under the Gaussian and Poissonian observation noises.

## 1. Introduction

In the field of medical imaging and noninvasive measurement, computed tomography (CT) plays an important role in diagnosis. The tomography image is reconstructed from a series of projection data, which are transmitted signals throughout an object, such as X-rays, in multiple directions. A lot of algorithms have been proposed to reconstruct tomography images [1–4]. Radon transform is usually used in mathematical formulations to describe the generating process of the observation data, and inverse of the Radon transform is considered as one of the frameworks for the image reconstruction from observation data; unfortunately, this reconstruction formulation does not care about noisy observations.

In order to improve image quality occurred by noisy observation, several image restoration methods based on the Bayesian inference are discussed in the field of image processing [5, 6]. The purpose of image restoration lends itself naturally to the Bayesian formulation, which infers a posterior probability for the original image using the prior probability of an assumed model for the original image and the corruption process. One well-known strategy for Bayesian image restoration is to adopt the image that maximizes the posterior probability; this is called the maximum a posteriori (posterior) probability (MAP) inference. In MAP inference, the quality of a restoration image is controlled by the strengths ratio between fidelity of the observation process and the prior strength of the model. Hyperparameters are often introduced to describe these strengths of the ratio; however, these hyperparameters inference is a hard problem in the MAP framework. In order to estimate hyperparameters in the MAP framework, the cross-validation method is considered as effective; however, we consider that there exists several problems. The first point is computational cost. In general, the cross-validation requires high computational cost. And the second point is to determine the cost function for the hyperparameters. In the field of image restoration, several types of methods are compared [7]; however, it is difficult to choose a cost function that is suitable for our problem.

In contrast, from the viewpoint of the Bayesian inference, the hyperparameter inference problem can be expressed naturally. For example, in the field of the image restoration, Molina et al. demonstrated several hyperparameter inference methods in the Bayesian manner in the manner of a hierarchical Bayes inference [8]. Pryce and Bruce and MacKay et al. proposed marginal likelihood maximization to infer those hyperparameters, which is called “evidence” framework or type 2 marginal likelihood maximization [6, 9–13].

In typical conventional methods, which use MAP inference for the computed tomography, a cost function that consists of data-fitting terms and several smoothness constraints has been introduced, and a minimization of the cost function is carried out in order to obtain the reconstructed image from the noisy observation data. Unfortunately, there have been few discussions related to the inference of a proper ratio between the data fitting and the constraints within the MAP framework. On the contrary, from the Bayesian inference point of view, it is natural to discuss the hyperparameter inference for image restoration using an evidence framework [14–16].

In our previous work, we proposed a CT image reconstruction in the manner of Bayes inference with a hyperparameter inference method from the noisy Radon-transformed observation by the evidence framework [12, 13]. In the previous work [12], however, we only showed that the Bayesian inference framework works well in the specific environment, that is, we assumed the additive white Gaussian noise for the 2-dimensional object observation. Gaussian noise is one of the tractable models for a mathematical formulation; however, in the X-ray CT or positron emission tomography (PET) image observation, we should assume Poissonian noise for the observation. Thus, in this study, we show that our reconstruction algorithm also works well under the Poissonian noise as well as under the Gauss noise case. Considering the Poissonian noise case for the observation which is different from our assuming model, we show a kind of robustness of our reconstruction model.

Moreover, we apply our reconstruction model into the real CT image data. Shepp and Logan phantom, which is usually used for evaluation of CT/PET image reconstruction, is a simple model of the axial cross-section human body. The internal organ of human body is not so much simple, so we use a real CT image data for reconstruction.

## 2. Formulation

In order to explain our Bayesian inference method, we show the conventional CT reconstruction method using filtered backprojection (FBP) under the formulation of the Radon transform. After that, we introduce Bayesian inference into the reconstruction process.

### 2.1. Radon Transform

**Figure 1:**
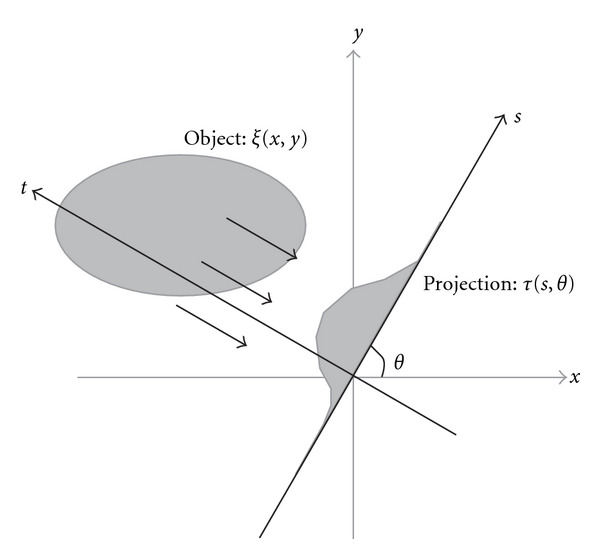
Schematic diagram of the Radon transform. Detectors are aligned on the *s* axis, which has an angle described as *θ*.

Briefly, the Radon transform assumes that the observed signals are transmitted through the target object.  Figure 1 shows the schematic diagram of the Radon transform. We describe the target object density as the function of the (*x*, *y*) coordinate and assume that the detectors are aligned along the *s* axis that is rotated in *θ*. We can thus denote the relationship between the (*x*, *y*) and (*s*, *t*) coordinates as a rotation

(1) $$\left(\begin{array}{c} s\\ t \end{array}\right)=\left(\begin{array}{cc} \cos\theta & \sin\theta \\ -\sin\theta & \cos\theta \end{array}\right)\left(\begin{array}{c} x\\ y \end{array}\right).$$

We describe the density of the target as *ξ*(*x*, *y*, *z*), that is, *ξ*(*x*, *y*, *z*) represents the absorption coefficients of the X-ray in the case of X-ray CT observation. The detectors are aligned on the *s* axis, so we describe the observation *τ*(*s*, *θ*, *z*) as the following formulation, called Radon transform:

(2) τ(s,θ,z)=∫dtξ(x,y,z)=∫dtξ(x(s,t),y(s,t),z).

### 2.2. FBP Reconstruction

Before introducing the Bayes inference, we formulate the conventional filtered backprojection (FBP) method. This reconstruction method is mainly formulated on the frequency domain, so we introduce the 2-dimensional Fourier transform of the reconstruction image *σ*(*x*, *y*) and its inverse transform pair as

(3) $$\begin{array}{c} \tilde{\sigma }\left(\tilde{x},\tilde{y}\right)={∭}_{}^{}dx\;dy\;\sigma \left(x,y\right)e^{-2\pi j(x\tilde{x}+y\tilde{y})}, \end{array}$$

(4) $$\begin{array}{c} \sigma \left(x,y\right)=\iint_{}^{}d\tilde{x}\;d\tilde{y}\;\tilde{\sigma }\left(\tilde{x},\tilde{y}\right)e^{2\pi j(x\tilde{x}+y\tilde{y})}, \end{array}$$

where the $\left(\tilde{x},\tilde{y}\right)$ represents the frequency space coordinate.

Meanwhile, we can apply a 1-dimensional Fourier transform for the *s* of the observed data *τ*(*s*, *θ*) as $\tilde{\tau }\left(\tilde{s},\theta \right)$. The $\tilde{\tau }\left(\tilde{s},\theta \right)$ satisfies the following relationship, which is called a projection theorem:

(5) $$\tilde{\tau }\left(\tilde{s},\theta ,\tilde{z}\right)=\tilde{\xi }\left(\tilde{s}\cos\theta ,\tilde{s}\sin\theta ,\tilde{z}\right).$$

The FBP method is derived as a coordinate transformation from Cartesian coordinate $\left(\tilde{x},\tilde{y}\right)$ into the polar coordinate $\left(\tilde{s},\theta \right)$ in the inverse Fourier transform (4)

(6) $$\begin{array}{cc} \sigma \left(x,y,z\right) & =\overset{\pi }{\underset{0}{\int }}d\theta \overset{\infty }{\underset{-\infty }{\int }}d\tilde{s}\left|\tilde{s}\right|\tilde{\sigma }\left(\tilde{s}\cos\theta ,\tilde{s}\sin\theta \right)e^{2\pi js\tilde{s}} \end{array}$$

(7) $$\begin{array}{cc} & =\overset{\pi }{\underset{0}{\int }}d\theta g\left(s,\theta \right), \end{array}$$

where

(8) $$g\left(s,\theta \right)=\int d\tilde{s}\left|\tilde{s}\right|\tilde{\tau }\left(\tilde{s},\theta \right)\;e^{2\pi js\tilde{s}},$$

since we can assume that the reconstruction image *σ*(*x*, *y*) should be identical to the original image *ξ*(*x*, *y*) without the observation noise, and we can apply the projection theorem in (5).

Thus, the reconstructed image *σ*(*x*, *y*) can be obtained by substituting the coordinate relationship *s* = *x*cos⁡*θ* + *y*sin⁡*θ*, that is derived from the rotation coordinate in (1) into (7). We call this reconstruction method the FBP method [1, 2].

### 2.3. Stochastic Model

In this section, we introduce a stochastic observation process into the FBP method. Of course, it is natural to consider Poissonian noise for observation in a realistic model; however, introducing Poissonian process makes it hard to solve the reconstruction in analytic form. We consider that a solvable model is important for understanding the reconstruction process. So in our theoretical framework, we introduced additive white Gaussian noise for observation on the signal *ξ*(*x*, *y*). When we consider the Gaussian noise *n*_*p*_(*x*, *y*) on the image *ξ*(*x*, *y*), the observation through the Radon transform *τ*(*s*, *θ*) can be described as

(9) $$\tau \left(s,\theta \right)=\int dt\left(\sigma \left(x,y\right)+n_{p}\left(x,y\right)\right)=\int dt\sigma \left(x,y\right)+N_{p}\left(s,\theta \right),$$

where *N*_*p*_(*s*, *θ*) = ∫*dt* *n*_*p*_(*x*, *y*), and we also treat it as Gaussian noise. In the manner of the conventional image restoration method proposed by Tanaka and Inoue, we also introduce the energy function *H*_*n*_(*τ* | *σ*) as follows [14, 16]:

(10) $$H_{n}\left(\tau \;|\;\sigma \right)=4\pi^{2}\overset{\pi }{\underset{0}{\int }}d\theta \int ds{\left(\tau \left(s,\theta \right)-\int dt\sigma \left(x,y\right)\right)}^{2}.$$

The important point of (10) is that the energy function *H*_*n*_(*τ* | *σ*) is defined as a kind of quadrature form of the difference between observation *τ*(*s*, *θ*) and the Radon transform of the reconstruction image ∫*dtσ*(*x*, *y*). We can thus denote the observation process as

(11) $$\begin{array}{c} p\left(\tau \;|\;\sigma \right)=\frac{1}{Z_{n}\left(\gamma \right)}\exp\left(-\gamma H_{n}\left(\tau \;|\;\sigma \right)\right), \end{array}$$

(12) $$\begin{array}{c} Z_{n}\left(\gamma \right)=\underset{\tau }{\sum }\exp\left(-\gamma H_{n}\left(\tau \;|\;\sigma \right)\right), \end{array}$$

where *Z*_*n*_(*γ*) is to normalize a factor called the partition function. The hyperparameter *γ* represents a precision parameter that is proportionate to the inverse of the variance of the Gaussian noise *N*_*p*_(*s*, *θ*), that is, the large *γ* indicates a good S/N ratio in the observation. Moreover, introducing both a polar coordinate for the frequency domain and Planchrel's theorem, we can drive the following expression:

(13) $$p\left(\tau \;|\;\sigma \right)=\frac{1}{Z_{n}\left(\gamma \right)}\exp\left(-4\pi^{2}\gamma \int d\theta \int d\tilde{s}\;{\left|{\tilde{\tau }}_{\tilde{s},\theta }-{\tilde{\sigma }}_{\tilde{s},\theta }\right|}^{2}\right),$$

where ${\tilde{\tau }}_{\tilde{s},\theta }=\tilde{\tau }\left(\tilde{s},\theta \right)$ and ${\tilde{\sigma }}_{\tilde{s},\theta }=\tilde{\sigma }\left(\tilde{s}\cos\theta ,\tilde{s}\sin\theta \right)$. In the following formulation, we adopt these expressions for the polar coordinate in the frequency domain description for the sake of convenience.

To reconstruct an image from noisy data, using Bayes inference, we also denote the prior distribution. At first, we introduce the following energy function *H*_MRF_(**σ**) for smoothness of the image:

(14) $$H_{\text{MRF}}\left(\sigma \right)=\iint_{}^{}dx\;dy\;{||\nabla \sigma (x,y)||}^{2},$$

where ∇ means gradient operator ∇ = (∂/∂*x*, ∂/∂*y*). This energy plays a role in the Markov random field (MRF) like a constraint since the gradient operation in the discretized space can be regarded as the difference between the neighboring pixels. So, this constraint controls neighboring pixel values to become similar to the target pixel. Then, we also introduce the following energy constraint to avoid taking large absolute pixel values:

(15) $$H_{L2}\left(\sigma \right)=\iint_{}^{}dx\;dy{||\sigma \left(x,y\right)||}^{2},$$

which are sometimes called “*L*_2_ constraint.” Hence, we treat the prior as Gibbs-Boltzmann distribution of the linear combination of energies *H*_MRF_(*σ*) and *H*_*L*2_(*σ*)

(16) $$\begin{array}{c} p\left(\sigma \right)=\frac{1}{Z_{\text{pri}}\left(\beta ,h\right)}\exp\left(-\beta H_{\text{MRF}}\left(\sigma \right)-4\pi^{2}hH_{L2}\left(\sigma \right)\right), \end{array}$$

(17) $$\begin{array}{c} Z_{\text{pri}}\left(\beta ,h\right)=\underset{\sigma }{\sum }\exp\left(-\beta H_{\text{MRF}}\left(\sigma \right)-4\pi^{2}hH_{L2}\left(\sigma \right)\right). \end{array}$$

The hyperparameters *β* and *h* control the strength of each constraint. The prior probability can thus be described as follows when we adopt the polar coordinate in the frequency domain:

(18) $$p\left(\sigma \right)=\frac{1}{Z_{\text{pri}}\left(\beta ,h\right)}\exp\left(-4\pi^{2}\overset{}{\underset{}{\int }}d\theta \overset{}{\underset{}{\int }}d\tilde{s}\left(\beta {\tilde{s}}^{2}+h\right)\left|\tilde{s}\right|{\left|{\tilde{\sigma }}_{\tilde{s},\theta }\right|}^{2}\right).$$

From (13) and (18), we can derive the posterior probability with Bayes theorem *p*(*τ* | *σ*) = *p*(*τ* | *σ*)*p*(*σ*)/∑_*σ*_*p*(*τ* | *σ*)*p*(*σ*). Then, we can describe the posterior as

(19) $$p\left(\tau \;|\;\sigma \right)\propto \exp\left(-4\pi^{2}\overset{\pi }{\underset{0}{\int }}d\theta \overset{}{\underset{}{\int }}d\tilde{s}\;F_{\tilde{s}}{\left|{\tilde{\sigma }}_{\tilde{s},\theta }-\frac{\gamma }{F_{\tilde{s}}}{\tilde{\tau }}_{\tilde{s},\theta }\right|}^{2}\right),$$

where $F_{\tilde{s}}=\left(\beta {\tilde{s}}^{2}+h\right)\left|\tilde{s}\right|+\gamma$.

In order to calculate the denominator value called partition function, we discretize the integral description in the partition function over polar coordinate in frequency domain. When we denote the sampling width for radial direction and polar angle as $\Delta_{\tilde{s}}$ and Δ_*θ*_, respectively, the discretized sampling point $\left({\tilde{s}}_{\tilde{k}},\theta_{l}\right)$ can be described as ${\tilde{s}}_{\tilde{k}}=\tilde{k}\Delta_{\tilde{s}}$ and *θ*_*l*_ = *l*Δ_*θ*_, respectively, where $\tilde{k}$ and *l* represent the indexes of the radial direction and the polar angle. The angle *θ*_*l*_ corresponds to the detectors array angle in the observation. Therefore, we assume that the observation is carried out *N*_*θ*_ times in the angle [0, *π*], that is, Δ_*θ*_ = *π*/*N*_*θ*_. The coordinate value ${\tilde{s}}_{\tilde{k}}$ represents the position in the radial direction, which means the spatial frequency described in the Fourier transform. From the Nyquist frequency, we can denote $\Delta_{\tilde{s}}=1/N_{s}\Delta_{s}$, where Δ_*s*_ is an interspace of the detectors in the array. We assume the length of detectors array as *L*, and *N*_*s*_ detectors are assigned with the same interspace in the array, so Δ_*s*_ = *L*/*N*_*s*_.

When we discretize the integral $\int d\tilde{s}$ in the posterior as $\sum_{\tilde{k}}^{N_{s}-1}\Delta_{\tilde{s}}$, we can derive the marginalized posterior probability as a Gaussian distribution

(20) $$p\left({\tilde{\sigma }}_{\tilde{k},l}\;|\;\tau \right)=𝒩\left({\tilde{\sigma }}_{\tilde{k},l}\;|\;\frac{\gamma }{F_{\tilde{k}}}{\tilde{\tau }}_{\tilde{k},l},\frac{N_{s}}{8\pi^{2}\Delta_{\theta }\Delta_{s}F_{\tilde{k}}}\right),$$

where the descriptions ${\tilde{\sigma }}_{\tilde{k},l}$, ${\tilde{\tau }}_{\tilde{k},l}$, and $F_{\tilde{k}}$ represent ${\tilde{\sigma }}_{\tilde{k},l}=\tilde{\sigma }\left({\tilde{s}}_{\tilde{k}}\cos\theta_{l},{\tilde{s}}_{\tilde{k}}\sin\theta_{l}\right)$, ${\tilde{\tau }}_{\tilde{k},l}=\tilde{\tau }\left({\tilde{s}}_{\tilde{k}},\theta_{l}\right)$, and $F_{\tilde{k}}=F_{{\tilde{s}}_{\tilde{k}}}=\left(\beta {\tilde{s}}_{\tilde{k}}^{2}+h\right)\left|{\tilde{s}}_{\tilde{k}}\right|+\gamma$, respectively.

### 2.4. Image Reconstruction

We adopt the marginalized posterior mean 〈*σ*(*x*, *y*)〉 for the image reconstruction solution. The posterior mean can be denoted as

(21) $$\langle \sigma \left(x,y\right)\rangle =\overset{\pi }{\underset{0}{\int }}d\theta \overset{\infty }{\underset{-\infty }{\int }}d\tilde{s}\left|\tilde{s}\right|\langle {\tilde{\sigma }}_{\tilde{s},\theta }\rangle e^{2\pi j\tilde{s}(x\cos\theta +y\sin\theta )}.$$

Thus, $\left\{\langle {\tilde{\sigma }}_{\tilde{s},\theta }\rangle \right\}$, which represents an average set of Fourier expressions, is required to obtain the mean pixel value over the posterior 〈*σ*(*x*, *y*)〉. We can evaluate $\langle {\tilde{\sigma }}_{\tilde{s},\theta }\rangle$ by discretizing the coordinate as in the previous section, thereby obtaining

(22) $$\langle {\tilde{\sigma }}_{\tilde{k},l}\rangle =\frac{\gamma }{F_{\tilde{k}}}{\tilde{\tau }}_{\tilde{k},l}.$$

This solution, called the posterior mean (PM) solution, provides identical result as the MAP does, that is, energy function *H*_*n*_(*σ*) minimization with the constraint of the smoothness of *H*_MRF_(*σ*) and *H*_*L*2_(*σ*),

(23) $$\begin{array}{cc} \sigma_{\text{MAP}} & =\text{argma}{\text{x}}_{\sigma }\ln\text{p}\left(\tau \;|\;\sigma \right)p\left(\sigma \right)\\ & =\text{argmi}{\text{n}}_{\sigma }(4\pi^{2}\gamma H_{n}\left(\tau \;|\;\sigma \right)\\ & \;+\beta H_{\text{MRF}}\left(\sigma \right)+4\pi^{2}hH_{L2}\left(\sigma \right)). \end{array}$$

Of course, PM solution is not identical to MAP solution in general; however, in this case, the PM solution and the MAP solution are identical, because the posterior distribution is denoted as a Gaussian distribution.

### 2.5. Hyperparameter Inference

To reconstruct an appropriate tomography image with our Bayesian inference, we need to assign proper values to the hyperparameters *β*, *h*, and *γ*. These hyperparameters *β* and *h* control the strength of constraints, while *γ* controls the fidelity of the observation. We infer these hyperparameters by using maximization of marginal log likelihood, which is sometimes called evidence framework [9–11]. The marginal log-likelihood is denoted as the linear combination of log partition functions,

(24) $$\ln p\left(\tau \;|\;\beta ,h,\gamma \right)=\ln Z_{\text{post}}\left(\beta ,h,\gamma \right)-\ln Z_{\text{n}}\left(\gamma \right)-\ln Z_{\text{pri}}\left(\beta ,h\right),$$

where *Z*_n_(*γ*) is also denoted as (12), *Z*_pri_(*β*, *h*) is denoted as (17), and, for the posterior, we introduce *Z*_post_(*β*, *h*, *γ*);

(25) $$\begin{array}{cc} Z_{\text{post}}\left(\beta ,h,\gamma \right) & =\underset{\sigma }{\sum }\exp(-4\pi^{2}\gamma H_{\text{n}}\left(\tau \;|\;\sigma \right)\\ & \;-\beta H_{\text{MRF}}\left(\sigma \right)-4\pi^{2}hH_{\text{L}2}\left(\sigma \right)). \end{array}$$

We use discretization to evaluate each partition function and obtain

(26) $$\begin{array}{cc} \ln Z_{\text{pri}}\left(\beta ,h\right) & =-\frac{N_{\theta }}{2}\overset{N_{s}-1}{\underset{\tilde{k}=0}{\sum }}\ln\left(\beta {\tilde{s}}_{\tilde{k}}^{2}+h\right),\\ \ln Z_{\text{n}}\left(\gamma \right) & =-\frac{N_{\theta }N_{s}}{2}\ln\gamma ,\\ \ln Z_{\text{post}}\left(\beta ,h,\gamma \right) & =-\frac{4\pi^{2}\Delta_{\theta }\Delta_{s}}{N_{s}}\overset{N_{s}-1}{\underset{\tilde{k}=0}{\sum }}\gamma \left(1-\frac{\gamma }{F_{k}}\right)\\ & \;\times {\left|\tau_{\tilde{k},l}\right|}^{2}-\frac{N_{\theta }}{2}\overset{N_{s}-1}{\underset{\tilde{k}=0}{\sum }}\ln F_{\tilde{k}}. \end{array}$$

To maximize the marginal log likelihood (24), we adopt a naive gradient method corresponding to the hyperparameters *β*, *h*, and *γ*, that is, we update hyperparameters using the following rule:

(27) $$\left(\begin{array}{c} \ln\beta^{t+1}\\ \ln h^{t+1}\\ \ln\gamma^{t+1} \end{array}\right)=\left(\begin{array}{c} \ln\beta^{t}\\ \ln h^{t}\\ \ln\gamma^{t} \end{array}\right)+\eta \left(\begin{array}{c} \frac{\partial \ln p\left(\tau \;|\;\beta^{t},h^{t},\gamma^{t}\right)}{\partial \ln\beta }\beta^{t}\\ \frac{\partial \ln p\left(\tau \;|\;\beta^{t},h^{t},\gamma^{t}\right)}{\partial \ln h}h^{t}\\ \frac{\partial \ln p\left(\tau \;|\;\beta^{t},h^{t},\gamma^{t}\right)}{\partial \ln h}\gamma^{t} \end{array}\right),$$

where *η* is a sufficiently small value. Those update rules (27) are denoted for ln⁡*β*, ln⁡*h*, and ln⁡*γ*, since *β*, *h*, and *γ* should be nonnegative values.

## 3. Evaluation by a Computer Simulation

### 3.1. Phantom Image Reconstruction

In the computer simulation, we created the Shepp and Logan phantom image in *N*_*x*_×*N*_*y*_ (pixels) and mapped the image into an origin-centered square with an edge length set to *L*, that is, the area is set to [−*L*/2, −*L*/2] × [*L*/2, *L*/2]. In the square, the area, which takes distance from the origin larger than *L*/2, is sometimes unobservable by the detectors from several angles, and we therefore ignore this area during our evaluation. For each angle *θ*_*l*_, we assume the *s* axis as Figure 1, and the origin in the (*x*, *y*) coordinate projects to the point *s* = 0 in any angle. We set the sampling parameters as *N*_*x*_ = *N*_*y*_ = *N*_*θ*_ = *N*_*s*_ = 256, and the length of the detectors array as *L* = 1.

For hyperparameter inference, we adopt a gradient method that requires initial state of these parameters. In the following simulations, the initial state of *β*^(0)^, *h*^(0)^, and *γ*^(0)^ is set to be 1. And the number of iterations is limited to the 10000 times.

#### 3.1.1. Gaussian Noise Case

In order to evaluate the performance of the hyperparameter inference, we carry out the simulation in the additive white Gaussian noise environment at first. We assumed that the Gaussian noise *n*_*p*_(*x*, *y*) was added during the observation process (see (9)) and controlled the noise standard deviation (SD) in the range of 0 to 6. A small SD means the low noise level in the observation process, and the larger SD becomes, the higher additive Gaussian noise level becomes. On the other hand, the large SD observation makes a lot of information loss for reconstruction. The MRF like prior (16) plays a roll of compensation for the information loss. In the simulation, Gaussian noise value sometimes makes fluctuation to the result, so we evaluated the average performance over 10 trials.

The computational cost is mainly consumed by hyperparameters inference. In this study, we adopted gradient method for the hyperparameter inference, so the computational cost depends on the initial state of these hyperparameters and learning coefficients *η*. In typical cases, about 1000~2000 iterations are required to converge for the *η* = 10^−6^. It takes 1~2 minutes for Intel Xeon E5530 2.40 GHz with 24 GiB memory.

**Figure 2:**
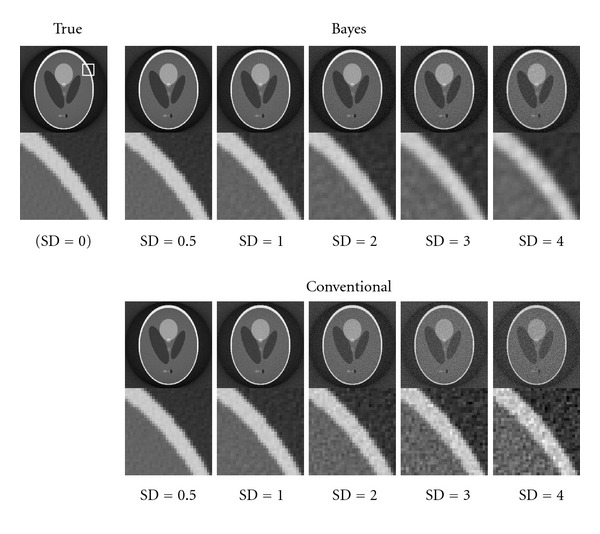
Comparison of the reconstructed tomography images derived using the Bayesian method and conventional FBP. The top row shows the Bayesian FBP methods, and the bottom one shows the conventional one. Each column corresponds to the strength of the observation Gaussian noise standard deviations. We show the magnification of a part of the reconstructed images around the edge of the phantom, whose location is indicated by white rectangle in the true image.

Figure 2 shows typical results of the reconstruction images. The most left image shows the “true” which means a reconstruction image without any observation noise (SD = 0.0). The top part shows the result using our Bayesian inference with inferred hyperparameters, and the bottom one shows the result using the conventional FBP method [1]. Each column corresponds to the SD of the additive Gaussian noise *n*_*p*_(*x*, *y*). In Figure 2, we show magnification of each reconstructed image around the edge whose location is located as the white rectangle in the “true” image. The degradation of the image in the conventional FBP result when the noise SD is large is clearly visible, whereas the contrast of the image has been maintained in the Bayesian reconstruction result.

**Figure 3:**
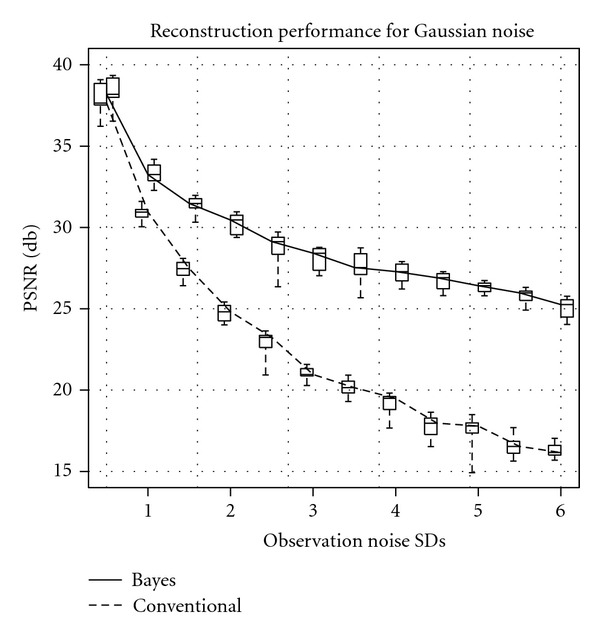
Qualities of reconstruction images measured by PSNR. The horizontal axis shows the SD of the Gaussian noise. The vertical axis shows the PSNR. The solid line shows the median of the 10 trials of our Bayesian inference results, and box plot shows quartile deviation. The dashed line shows the results of the conventional FBP method.

We used the peak signal-to-noise ratio (PSNR) to evaluate the quality of the reconstructed image. The result of this evaluation is shown in Figure 3. The horizontal axis indicates the SD of the *n*_*p*_(*x*, *y*), and the vertical shows the PSNR between the reconstructed image for both a noised and noiseless reconstruction images. The solid line shows the median of the Bayesian inference reconstruction results for 10 trials, and each box plot shows the quartiles deviations. The dashed one shows those of the conventional FBP results. The Bayesian inference maintained high reconstruction quality compared to the conventional FBP method. Even when the SD of the noise was 4.0, the PSNR value remained 27.5 (dB). On the other hand, the PSNR of the conventional FBP method was degraded and became 27.7 [dB] when the SD is only 1.5. This demonstrated that the Bayesian inference is more robust to the observation noise rather than the conventional FBP method.

**Figure 4:**
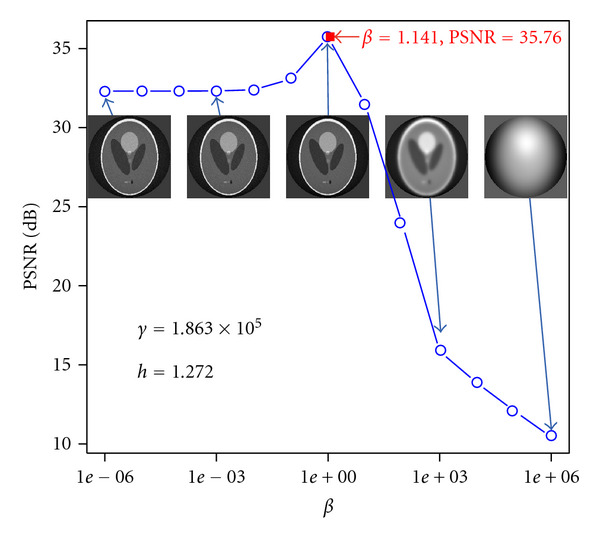
Reconstruction performance against hyperparameter *β*. The horizontal axis shows the *β*, and the vertical one shows the PSNR. Other hyperparameters, *h* and *γ*, are fixed to the estimated value. The filled rectangle shows the result of our hyperparameter inference for *β*.

Figure 4 shows the reconstruction performance against the hyperparameter *β*. The horizontal axis shows the value of the hyperparameter *β*, and the vertical one shows the PSNR. We fixed other hyperparameters, *h* and *γ*, to the estimated value. Each image in the figure shows the reconstruction result with corresponding hyperparameter setting. The hyperparameter *β* controls the smoothness of the image in the prior equation (16), so too much large *β* makes excessive blurring. Our hyperparameter inference algorithm, shown in the filled rectangle in the figure, looks to provide optimal value.

#### 3.1.2. Poissonian Noise Case

Gauss noise observation is the assumed model in our formulation equation (9); however, the CT/PET observation process is usually described as the Poissonian process. Thus, we should evaluate the reconstruction quality for the Poissonian noise case for the more realistic environment. Of course, our model is designed for the Gaussian noise case, so the performance of reconstruction for the Poissonian process observation might become worse; however, quantitative evaluation is important in the meaning of the approximation.

In the computer simulation, we used R PET package for Poissonian noise sampling [17]. The Poissonian noise value is generated by acceptance-rejection method [18, 19]. Hence, the number of the samplings determines the noise strength property corresponding to the SDs in the Gaussian case, that is, less number of the samplings make low signal-to-noise ratio. The computational cost is also consumed by hyperparameter inference, and it takes about 1000 times iterations for the *η* = 10^−6^, that is, it requires ~1 minute for the convergence in our computational environment.

**Figure 5:**
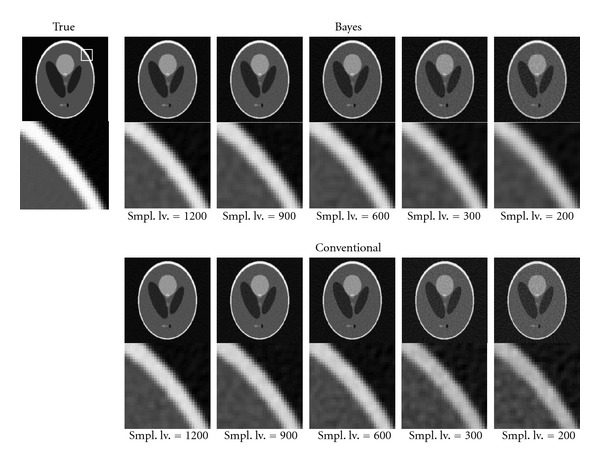
Comparison of the reconstructed tomography images derived using the Bayesian method and conventional FBP under the Poissonian noise. The top row shows the results of our method, and the bottom one shows the results of the conventional FBP method. Each column corresponds to the strength of the observation noise which can be denoted as the number of sampling in the acceptance rejection method. We show the magnification of a part of the reconstructed images around the edge of the phantom, whose location is indicated by a white rectangle in the true image.

Figure 5 shows the reconstructed image using our Bayesian method and conventional FBP method. The top part shows the result of our Bayesian reconstruction images, and the bottom one shows the conventional FBP result. The most left image shows also the “true” image that means a reconstructed image without any Poissonian noise. In other columns, we show the image with Poissonian noise whose strength is controlled by sampling levels, that is, the S/N ratio becomes worse when sampling level becomes low [20]. In the figure, the noise strength becomes large for the right direction.

**Figure 6:**
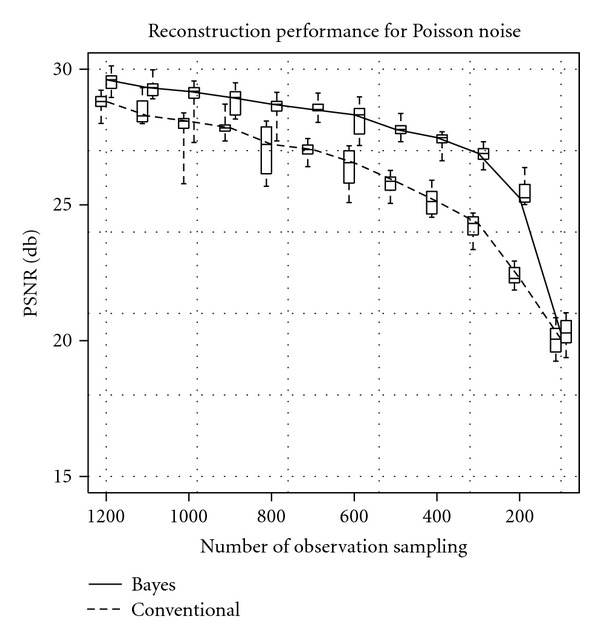
Qualities of reconstruction images measured by PSNR. The horizontal axis shows the SD of the sampling level, which means the inverse of the Poissonian noise. The vertical axis shows the PSNR. The solid line shows the median of the 10 trials of our Bayesian inference results, and box plot shows quartile deviation. The dashed line shows the results of the conventional FBP method.

Figure 6 shows the quantity evaluation result in the meaning of the PSNR against the sampling level of the observation. The horizontal axis shows the sampling level, and the vertical one shows the PSNR. The solid line shows the median of 10 trials for our Bayesian reconstruction method, and the box plots are quartiles for each sampling levels. The dashed one shows the result of the conventional FBP method. Roughly speaking, the Bayes reconstruction shows better result in the meaning of the PSNR.

### 3.2. Real CT Image Reconstruction

In order to evaluate the performance of our method for the CT/PET image, we applied our method to a real CT image reconstruction.

We prepare several real CT images provided by Tokushima University Hospital. The acquisition parameters of those HRCT images are as follows: Toshiba “Aquilion 16” is used for imaging device, and each slice image consists of 512 × 512 pixels, and pixel size corresponds to 0.546~0.826 mm; slice thickness is 1 mm. Thus, we set the sampling parameters as *N*_*x*_ = *N*_*y*_ = *N*_*θ*_ = *N*_*s*_ = 512.

In order to obtain noise-corrupted data *τ*, we simulate Gaussian and Poissonian noised observation for these CT images in the same manner with phantom images.

**Figure 7:**
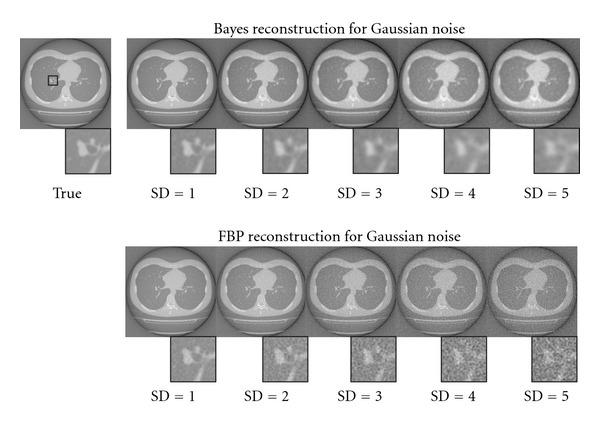
Comparison of the reconstructed images using real CT data with Gaussian noise between Bayes method and conventional FBP method. The top row shows the results of our method, and the bottom one shows the conventional FBP results. Each column corresponds to the strength of the observation noise that can be denoted as standard deviation (SD) of adding noise. We also show the magnification of a part around the bronchus, whose location is indicated by black rectangle in the true image.

**Figure 8:**
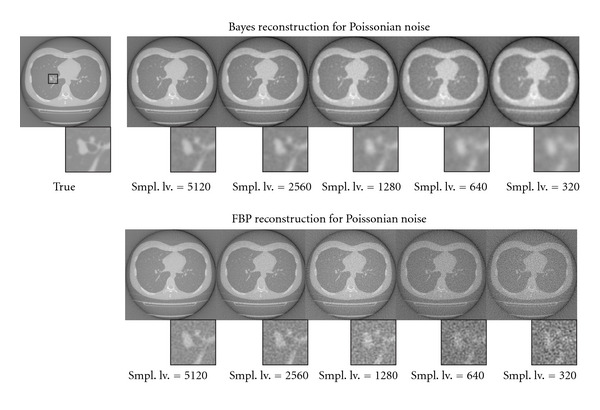
Comparison of the reconstructed images using real CT data with Poissonian noise between Bayes method and conventional FBP method. The top row shows the results of our method, and the bottom one shows the conventional FBP results. Each column corresponds to the strength of the observation noise that can be denoted as the number of sampling in the acceptance-rejection method. We also show the magnification of around bronchus indicated by black rectangle in the true image.

Figure 7 shows a reconstruction result for the real chest CT image with Gaussian noise. The top row shows our Bayesian method, and the bottom one shows the conventional FBP results. Each column corresponds to the additive Gaussian noise strength for pseudo-observation. In each image, we show a magnification part around bronchus, whose location is described as a black rectangle in the true image. In the Bayesian reconstruction, our MRF prior makes a blurring effect for edge components on the image. The hyperparameter inference mechanism would try to compensate for the information loss, which is caused by the observation noise, by use of the MRF prior. As a result, the large SD makes strong blurring effect to the image. In the magnification image of the Bayesian inference, the bronchus parts are hard to identify around SD > 4.0, however, vessels along the bronchus are able to identify for these SDs. In contrast, in the conventional FBP results, both of those parts are just difficult to identify for these SDs.  Figure 8 also shows a Poissonian noise case for the chest CT image. We can see the similar tendency to the Gaussian case. In the magnification images, we can identify the bronchus over 1280 sampling levels. In contrast, low sampling level makes large blurring effect by the MRF prior. As a result, bronchus part is hard to identify at fewer than 640 sampling levels. However, the reconstruction result looks better than those of the conventional FBP method.

**Figure 9:**
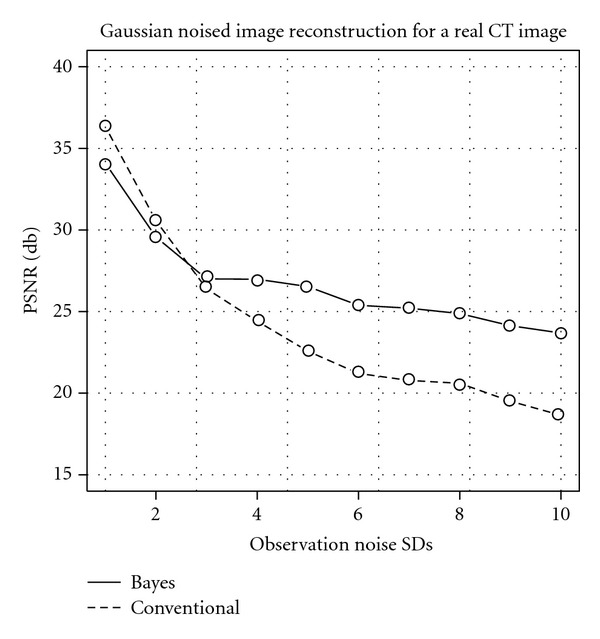
Qualities of reconstruction images measured by PSNR for real CT image reconstruction under the Gaussian noise. The horizontal axis shows the observation SD of Gaussian noise, and the vertical axis shows the PSNR. The solid line shows the result of our Bayesian method, and the dashed one shows that of the conventional FBP method.

**Figure 10:**
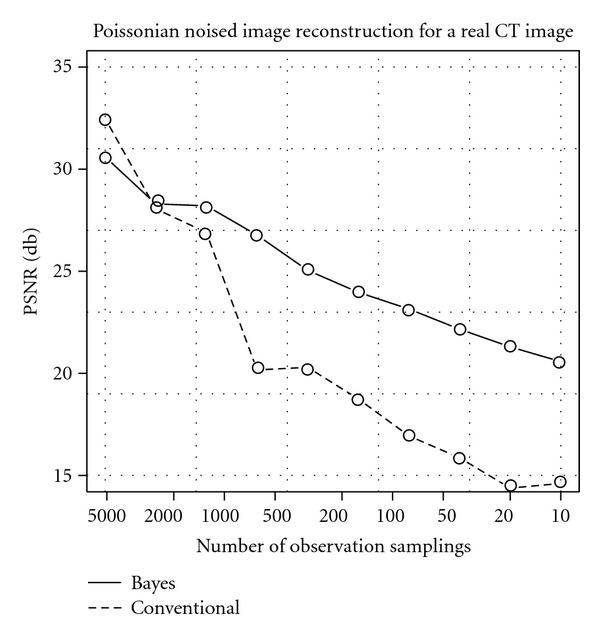
Qualities of reconstruction images measured by PSNR for real CT image reconstruction under the Poissonian noise. The horizontal axis shows the sampling level, and the vertical axis shows the PSNR. The solid line shows the result performance of Bayesian method, and the dashed line shows the results of the conventional FBP method.

Moreover, we evaluate the quantitative reconstruction performance by PSNR for the real CT image.  Figure 9 shows the result for the Gaussian noise case, and Figure 10 shows the one for the Poissonian case. Each horizontal axis means the noise strength control variable, and the vertical shows the reconstruction performance by PSNR. In both of these results, the Bayes reconstruction method shows better performances in the strong noise area. In contrast, in the weak noise area, the Bayes reconstruction result is just worse than that of the conventional method. We can see that the real CT image is more complex than the Shepp and Logan phantom image like Figure 5, and simple MRF like prior (16) prefers smooth image. Thus, in the weak noise area, complex shape in the real image makes overestimate for the prior strength *β*, which controls blurring effect by the prior. As a result, our Bayesian reconstruction method prefers too much smooth image in the weak noise area; however, the PSNR value stays around 30 (db) for the SD = 2 in the Gaussian case and around 28 (db) for the 2560 samplings in the Poissonian case.

## 4. Conclusion

We proposed a hyperparameter inference based on the Bayesian inference in order to reconstruct tomography image formulated by Radon transform. As a stochastic model, we introduced a simple MRF-like distribution *p*(*σ*) for the prior and formulated the observation process *p*(*τ* | *σ*) by assuming the Gaussian noise channel.

We discretized the image signals in the frequency domain expressed by the polar coordinate in order to evaluate the posterior distribution analytically, resulting in the ability to conduct posterior mean for the reconstructed image. Using the marginal-likelihood maximization method, we show that the hyperparameters introduced as *β*, *h*, and *γ*, which allows us to maintain a balance between observation fidelity and prior constraint, could be determined automatically. And using those hyperparameters, we could obtain a higher-quality reconstructed image than when using the conventional FBP method.

In order to evaluate the performance of our method, we simulated two observation noise cases, that is, Gaussian and Poissonian noises. We controlled noise strength by SD for Gaussian noise and sampling levels for Poissonian noise. In the phantom simulation for the Gaussian noise, we confirmed that our hyperparameter inference worked well against the PSNR, and the performance for the reconstruction was better than that of the conventional FBP. The computational cost for the hyperparameter inference depend on the initial state of them; however, about 1200~2000 times iterations made convergence to them for typical cases. In the Poissonian cases, the tendency of the reconstruction performance is similar to the Gaussian case. Our Bayesian method made better performance than the conventional FBP in any noise strength area. However, in the strong Poissonian noise case, that is, the noise could not approximate well by Gaussian noise, we confirmed that the performance of the reconstruction was not good enough for diagnosing. Moreover, we evaluated the performance by a real chest CT image. The real image has a little complex shape against the phantom image. Thus, in the low-noise strength area for both noise cases, the prior components worked too much for the smoothness effect. As a result, the PSNR was just worse than the conventional FBP in such area. However, detail structure of the organ was easy to identify in the obtained image of our model.

In this study, we demonstrate applying our algorithm to the only 2-dimensional image reconstruction. We consider the algorithm easy to extend for 3-dimensional case. Thus, we would reformulate our algorithm for applying to the 3-dimensional image reconstruction and confirm the performance in the future work.
